# A Novel Semi-Supervised Feature Extraction Method and Its Application in Automotive Assembly Fault Diagnosis Based on Vision Sensor Data

**DOI:** 10.3390/s18082545

**Published:** 2018-08-03

**Authors:** Xuan Zeng, Shi-Bin Yin, Yin Guo, Jia-Rui Lin, Ji-Gui Zhu

**Affiliations:** State Key Laboratory of Precision Measuring Technology and Instruments, Tianjin University, Tianjin 30072, China; zengxuan@tju.edu.cn (X.Z.); yin_guo@tju.edu.cn (Y.G.); linjr@tju.edu.cn (J.-R.L.); jiguizhu@tju.edu.cn (J.-G.Z.)

**Keywords:** automotive assembly, feature extraction, semi-supervised learning, complete kernel Fisher discriminant analysis, fault classification

## Abstract

The fault diagnosis of dimensional variation plays an essential role in the production of an automotive body. However, it is difficult to identify faults based on small labeled sample data using traditional supervised learning methods. The present study proposed a novel feature extraction method named, semi-supervised complete kernel Fisher discriminant (SS-CKFDA), and a new fault diagnosis flow for automotive assembly was introduced based on this method. SS-CKFDA is a combination of traditional complete kernel Fisher discriminant (CKFDA) and semi-supervised learning. It adjusts the Fisher criterion with the data global structure extracted from large unlabeled samples. When the number of labeled samples is small, the global structure that exists in the measured data can effectively improve the extraction effects of the projected vector. The experimental results on Tennessee Eastman Process (TEP) data demonstrated that the proposed method can improve diagnostic performance, when compared to other Fisher discriminant algorithms. Finally, the experimental results on the optical coordinate data proves that the method can be applied in the automotive assembly process, and achieve a better performance.

## 1. Introduction

As it is well-known, an automotive body is a complex structure consisting of a large amount of thin stamping sheets with different geometry shapes welded together. Dimensional variation produced during the welding process affects not only the appearance and performance of automotive products, but also the personal and property safety of the user. Thus, the fault diagnosis of dimensional variation is vital to the quality promotion of vehicles. In recent years, in order to meet the needs of different customer groups, automobile manufacturers must develop more detailed product lines, and inevitably, the product development cycle needed to be shortened. This has undoubtedly put forward higher requirements for fault diagnosis, including quick, high accuracy and intelligent.

The traditional coordinate data source is a coordinate measurement machine (CMM), which has been widely established in welding workshops to measure automotive body dimensional features [1]. Although CMM has high accuracy and reliability, it also has very obvious disadvantages, such as low efficiency and harsh environmental requirements. According to statistics, merely <3% of automotive bodies can be measured in most workshops [2], which is far from enough for quick fault discovery, much less for fault diagnosis. In recent years, with the rapid development of optical imaging devices, various vision-based systems have been widely applied in modern industries [3,4]. In automotive welding workshops, more and more hybrid visual inspection systems have been distributed on production lines for 100% online inspection [4]. Combined the industrial robot’s high-flexibility and high-speed visual inspection technology, the measure speed of a hybrid visual inspection system can reach 80 points per minute, which is much more efficient than CMM, and provides favorable conditions for data-driven fault diagnosis.

One of the important fault diagnosis methods for assembly dimensional variation is the model-based method, which achieves fault diagnosis by constructing a mathematical model, and estimating the residual between estimates and measurements. The classical model-based method is the state space modeling approach [5,6]. In this method, the dimensional variation propagation is described through a state space model, and an observation equation, which represents the relationship between the observation vector and state vector, is also established. The state space modeling provides a method to describe the overall body assembly variation propagation. However, once the number of welding stations increases, the modeling process will become extremely difficult, and even lead to failure of diagnosis. Different from model-based methods, the necessary process information of data-driven methods can be extracted directly from huge amounts of recorded process data. Hu was the first to introduce principal component analysis (PCA) into fixture fault diagnosis to extract the deviation pattern and identify the source of fault by comparing the deviation pattern with potential failure modes. Jang and Yang [7] adopted artificial neural networks (ANNs) to deal with noisy or missing data. Lian [8] extracted the variations of the measured data by correlation analysis, and found the root of fault through the maximal tree method. For small data set problems, Liu [2] established the Bayesian network model, and was successfully able to overcome it.

As a well-known feature extraction method, Fisher discriminant analysis (FDA) and its extension methods have been applied in various industries [9,10,11,12]. Chen [9] introduced local FDA and support vector machines for hepatitis disease diagnosis. Wen [10] applied weighted KFDA to extract the E-nose’s data, and achieved a desirable effect. Direct linear discriminant analysis was proposed for view-angle invariant gait recognition [11]. Unlike PCA, which is an unsupervised method, FDA takes full advantage of its sample label information, which can be regarded as a more outstanding method, compared to PCA [13]. However, these supervised methods need sufficient labeled samples to achieve a desirable result, which is hard to satisfy in the real world. Especially in the automobile industry, it is costly and time-consuming to label fault data with different causes. However, unlabeled data can be easily obtained. For example, if a fixture failure occurs in one assembly station, the corresponding dimension deviation will delay a period of time, which is difficult even for professional technicians to trace back.

In order to solve the problem of less labeled data, the investigators propose a novel semi-supervised complete kernel Fisher discriminant analysis method for fault diagnosis, called SS-CKFDA. The basic idea of SS-CKFDA is to extract the global structure from the unlabeled sample to enhance the generalization performance of FDA. Meanwhile, the kernel method is reserved to solve the nonlinear problem by projecting the data from the linear inseparable input space to a high-dimensional feature space, which is linearly separable. The innovative contributions of the present study are summarized, as follows:

(1) A novel semi-supervised feature extraction method called, SS-CKFDA, was proposed. In order to confirm the method’s effectiveness, a simulation experiment based on TEP data was performed, and the results were compared with three other FDA-based methods.
(2) SS-CKFDA was introduced to mine fault information obtained from the historical data of the hybrid visual inspection system. Based on this, a novel fault diagnosis flow was put forward for automotive body assembly.
(3) A real experimental system for the automotive body assembly process was realized, and the results show that the proposed method can greatly enhance diagnosis accuracy, especially when less labeled data are obtained.
(4) Two representative classifiers, k-nearest neighbor classifier (KNN) and minimum distance classifier (MD), were also discussed in the present study.

The present study is organized as follows: Section 2 presents the details of the proposed SS-CKFDA algorithm for fault diagnosis. The two different experimental datasets used in the present study are introduced in Section 3. In Section 4, simulations on TEP data were carried out to verify the effectiveness of the proposed SS-CKFDA algorithm, and analyze the effects of the parameters and classifiers. Then, the experimental results for the automotive assembly data were subsequently analyzed. Finally, the conclusion is presented in Section 5.

## 2. Methodology

### 2.1. Principle of the Complete Kernel Fisher Discriminant Analysis (CKFDA)

The CKFDA algorithm was proposed based on a novel framework, KPCA plus LDA, which makes full use of irregular discriminant information that exists in the null space of the within-class scatter matrix [14]. The irregular discriminant subspace, which is neglected in the previous KFDA algorithm, is as powerful as the regular discriminant subspace for feature recognition. Combined with discriminant vectors in regular and irregular subspaces, CKFDA can outperform other FDA-based algorithms. Although CKFDA was proposed as an effective tool for solving the “small sample size” problem, it has also been proven to be suitable for “large sample size” problems. The principle of CKFDA is as follows:

First, KPCA [15] is performed to transform the original input space ${ℜ}^{n}$ into a m-dimensional space ${ℜ}^{m}$. Hence, we first set forth the KPCA algorithm in detail. 

It is assumed that a set of M training samples $x_{1},x_{2},\ldots ,x_{M}$ in space ${ℜ}^{n}$ can be mapped into a feature space H by a given nonlinear mapping Φ:x↦Φ(x). Then, the covariance matrix on the feature space can be given by:

(1) $$S_{t}^{\Phi }=\frac{1}{M}\sum_{j=1}^{M}\left(\Phi (x_{j})-m_{0}^{\Phi }\right){\left(\Phi (x_{j})-m_{0}^{\Phi }\right)}^{T}$$

where $m_{0}^{\Phi }=\frac{1}{M}\sum_{j=1}^{M}\Phi (x_{j})$. In addition, for every eigenvector of $S_{t}^{\Phi }$, η can be linearly expanded by $\sum_{i=1}^{M}a_{i}\Phi (x_{i})$.

In order to obtain the expansion coefficients, $Q=\left[\Phi (x_{1}),\ldots \Phi (x_{M})\right]$ and the Gram matrix $\tilde{R}=Q^{T}Q$ were denoted, and the elements can be gained using the kernel trick:

(2) $${\tilde{R}}_{ij}=\Phi {\left(x_{i}\right)}^{T}\Phi (x_{j})=k(x_{i},x_{j})$$

Kernel function can be any function that fulfills Mercer’s condition [16]. The most common kernel functions are Gaussian kernel, polynomial kernel and sigmoid kernel.

Before the following operation, the Gram matrix $\tilde{R}$ should be centralized [16]. 

The orthonormal eigenvectors $\gamma_{1},\gamma_{2},\ldots ,\gamma_{m}$ of R are calculated, which correspond to the m largest eigenvalues $\lambda_{1}\geq \lambda_{2}\geq \ldots \lambda_{m}$. Then, eigenvector $\eta_{j}$ of $S_{t}^{\Phi }$ can also be obtained by:

(3) $$\eta_{j}=\frac{1}{\sqrt{\lambda_{j}}}Q\gamma_{j},\;j=1,\ldots ,m.$$

Lastly, the KPCA-transformed feature vector $y={\left(y_{1},y_{2},\ldots ,y_{m}\right)}^{T}$ can be obtained by:

(4) $$y=P^{T}\Phi (x),\;\mathrm{where}\;P=\left(\eta_{1},\eta_{2},\ldots ,\eta_{m}\right).$$

Next, LDA is performed in the KPCA-transformed space and the regular and irregular discriminant features are extracted. Suppose that there are c pattern classes, the between-class scatter matrix $S_{b}$ and the within-class scatter matrix $S_{w}$ in the KPCA-transformed space can be defined through the following pairwise forms [17]:

(5) $$S_{b}=\frac{1}{2}\sum_{i=1}^{n}\sum_{j=1}^{n}W_{i,j}^{b}\left(y_{i}-y_{j}\right){\left(y_{i}-y_{j}\right)}^{T}$$

(6) $$S_{w}=\frac{1}{2}\sum_{i=1}^{n}\sum_{j=1}^{n}W_{i,j}^{w}(y_{i}-y_{j}){\left(y_{i}-y_{j}\right)}^{T}$$

where:

(7) $$W_{i,j}^{b}=\{\begin{array}{cc} 1/n-1/n_{k} & \mathrm{if}\;y_{i}\in class\;k\;\mathrm{and}\;y_{j}\in class\;k\\ 1/n & \mathrm{otherwise} \end{array}$$

and:

(8) $$W_{i,j}^{w}=\{\begin{array}{cc} 1/n_{k} & \mathrm{if}\;y_{i}\in class\;k\;\mathrm{and}\;y_{j}\in class\;k\\ 0 & \mathrm{otherwise} \end{array}$$

$W_{i,j}^{b}$ and $W_{i,j}^{w}$ are the corresponding coefficient matrix of $S_{b}$ and $S_{w}$.

It was assumed that $\alpha_{1},\ldots ,\alpha_{m}$ are the orthonormal eigenvectors of $S_{w}$, and the first q ones correspond to positive eigenvalues, $q=rank(S_{w})$. Then, the irregular space of $S_{w}$ is the subspace $\Theta_{w}=span\left\{\alpha_{q+1},\ldots ,\alpha_{m}\right\}$, and its orthogonal complementary space $\Theta_{w}^{⊥}=span\left\{\alpha_{1},\ldots ,\alpha_{q}\right\}$ is the regular space.

In the regular space, $P_{1}=\left(\alpha_{1},\ldots ,\alpha_{q}\right)$, and the optimal regular mapping vectors can be gained by maximizing the Fisher criterion $\underset{\xi }{\arg\max}\frac{\xi^{T}{\tilde{S}}_{b}\xi }{\xi^{T}{\tilde{S}}_{w}\xi },\;\left(\xi \neq 0\right)$, where ${\tilde{S}}_{b}=P_{1}^{T}S_{b}P_{1}$, ${\tilde{S}}_{w}=P_{1}^{T}S_{w}P_{1}$ and ξ represents the optimal vectors of Fisher criterion. The optimization problem is proven to be equal to the generalized eigenvalue problem: ${\tilde{S}}_{b}\xi =\lambda {\tilde{S}}_{w}\xi$. After working out $u_{1},\ldots ,u_{d}\left(d\leq c-1\right)$, which correspond to the d largest positive eigenvalues of ${\tilde{S}}_{b}\xi =\lambda {\tilde{S}}_{w}\xi$, the optimal regular discriminant feature vector can be obtain, as follows:

(9) $$z^{1}=U^{T}P_{1}^{T}y,\;\mathrm{where}\;U=\left(u_{1},\ldots ,u_{d}\right)$$

In a similar way, in the irregular space, $P_{2}=\left(\alpha_{q+1},\ldots ,\alpha_{m}\right)$, and the criterion is converted into $\underset{\xi }{\arg\max}\xi^{T}{\hat{S}}_{b}\xi ,\;\left(\left\|\xi \right\|=1\right)$, where ${\hat{S}}_{b}=P_{2}^{T}S_{b}P_{2}$. The optimal vector $v_{1},\ldots ,v_{d}\left(d\leq c-1\right)$ of $\hat{J}(\xi )$ are the orthonormal eigenvectors of ${\hat{S}}_{b}$, which corresponds to d largest eigenvalues. Then, the optimal irregular discriminant feature vector can be obtained, as follows:

(10) $$z^{2}=V^{T}P_{2}^{T}y,\;\mathrm{where}\;V=\left(v_{1},\ldots ,v_{d}\right)$$

Finally, the two kinds of discriminant features are fused for classification. The normalized-distance between sample $z=\left[z^{1},z^{2}\right]$ and training samples $z_{i}=\left[z_{i}^{1},z_{i}^{2}\right]\;(i=1,\ldots ,M)$ is defined by:

(11) $$\bar{g}(z,z_{i})=\theta \frac{\left\|z^{1}-z_{i}^{1}\right\|}{\sum_{j=1}^{M}\left\|z^{1}-z_{j}^{1}\right\|}+\frac{\left\|z^{2}-z_{i}^{2}\right\|}{\sum_{j=1}^{M}\left\|z^{2}-z_{j}^{2}\right\|}$$

where θ is the fusion coefficient, which determines the weight of the regular discriminant information in the decision level. In this study, θ was set to 1.

To this point, the feature extraction process of CKFDA has been completed. The original features are reduced to two kinds of discriminant features $\left[z^{1},z^{2}\right]$, and the normalized-distance can be used for the next classification with one distance classifier.

### 2.2. Semi-Supervised Complete Kernel Fisher Discriminant Analysis

#### 2.2.1. Semi-Supervised Learning

Traditional supervised learning only uses labeled data to train, which inevitably tends to perform poorly due to overfitting when there are no adequate labeled samples. In some cases, such as automotive assembly quality monitoring, labeled samples are hard to obtain, while unlabeled data are abundant. Therefore, semi-supervised learning is an effective solution to reduce human labor and improve identification accuracy. The goal of semi-supervised learning is to change the learning behavior of the traditional supervised method by combining this with unlabeled data, and design algorithms that take advantage of such combination [18].

SELF is an effective graph based semi-supervised learning algorithm [17,18,19]. The basic idea of SELF is that the information extracted by limited labeled samples can be adjusted through the global structure of unlabeled samples. The algorithm flow of SELF is as follows.

Assuming that the input samples X can be expressed by $X=\left\{X_{L},X_{U}\right\}$, $X_{L}$ refers to labeled samples, which corresponds to the above $\left\{x_{i}^{j}\right\}$ with c classes, while $X_{U}$ refers to unlabeled samples. Then, SELF defines the regularized between-class and within-class scatter matrices, as follows:

(12) $$S_{rb}=\left(1-\beta \right)S_{b}+\beta S_{t}$$

(13) $$S_{rw}=\left(1-\beta \right)S_{w}+\beta I_{m}.$$

where $S_{b}$ and $S_{w}$ corresponds to the between-class scatter matrix and within-class scatter matrix of the labeled samples $X_{L}$ in Equations (5) and (6). $S_{t}=\frac{1}{2}\sum_{i=1}^{n}\sum_{j=1}^{n}W_{i,j}^{t}(x_{i}-x_{j}){\left(x_{i}-x_{j}\right)}^{T}$ corresponds to the total scatter matrix of all samples X, where $W_{i,j}^{t}=1/n$. β is a trade-off parameter, and its value range is [0,1]. 

In SELF, $S_{t}$ represents the global structure, and by adjusting parameter β, the influence of $S_{t}$ can be controlled. When β=0, SELF inclines to FDA, which is a supervised method, and when β=1, SELF deteriorates to PCA, which is an unsupervised method. When β∈(0,1), SELF is a semi-supervised method.

#### 2.2.2. The SS-CKFDA Algorithm

Based on the above idea of semi-supervised learning, a novel semi-supervised CKFDA algorithm (SS-CKFDA), which combined CKFDA with SELF, was proposed first. Although CKFDA can achieve an ideal performance with labeled samples, the investigators still wanted to obtain more information from the unlabeled samples. Through the algorithm description of CKFDA, it could be observed that the double discriminant subspaces (DDS), which irregularly and regularly spanned in the CKFDA feature spaces, were learned from the labeled samples, and this can be inaccurate when the labeled samples are insufficient. By complementing semi-supervised learning in the feature spaces, DDS can be corrected through sufficient unlabeled samples. This is the basic idea of SS-CKFDA. The steps for the proposed SS-CKFDA algorithm are presented, as follows:
**Algorithm 1.** (SS-CKFDA Algorithm):Step 1:KPCA is performed for both labeled and unlabeled samples. Data $X=\left\{X_{L},X_{U}\right\}$ in input space ${ℜ}^{n}$ is transformed to data $Y=\left\{Y_{L},Y_{U}\right\}$ in feature space ${ℜ}^{m}$. Step 2:SELF is performed in ${ℜ}^{m}$. First, between-class scatter matrices $S_{b}$ and within-class scatter matrices $S_{w}$ are constructed with data $Y_{L}$ by Equations (5) and (6). Then, the regular between-class scatter matrices $S_{rb}$ and regular within-class scatter matrices $S_{rw}$ are constructed with data $S_{b}$, $S_{w}$ and Y by Equations (12) and (13).Step 3:The $S_{rw}$’s orthonormal eigenvectors $\alpha_{1},\ldots ,\alpha_{m}$ are calculated, assuming that the first *q* (*q* = rank($S_{rw}$)) corresponds to the positive eigenvalues.Step 4:The regular discriminant feature $z^{1}$ is extract in regular space $\left\{\alpha_{1},\ldots ,\alpha_{q}\right\}$ by Equation (9) and irregular discriminant feature $z^{2}$ is extracted in irregular space $\left\{\alpha_{q+1},\ldots ,\alpha_{m}\right\}$ by Equation (10).Step 5:The regular and irregular discriminant features are fused using Equation (11) for classification.

The proposed SS-CKFDA algorithm has two advantages. On one hand, kernel methods have been intensively used to determine the nonlinear structure, which adopts nonlinear mapping to map the input data into an implicit feature space, where the data will obtain a nonlinear representation. In order to avoid implicit features computation, nonlinear mapping usually adopts kernel tricks, that is, the nonlinear dot product kernel in the kernel methods. On the other hand, SELF was applied in DDS. SELF can incorporate the global structure of unlabeled samples into Fisher criterion to overcome the overfitting problem induced by the lack of labeled samples. 

#### 2.2.3. Comparison of SS-CKFDA with Other FDA Algorithms

**Figure 1:**
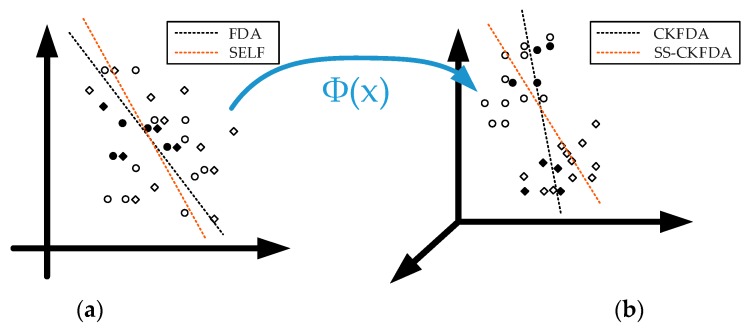
SS-CKFDA compared with other FDA-based methods, (**a**) input space, (**b**) fused feature space.

Figure 1 vividly describes the differences between SS-CKFDA and the other FDA algorithms using two class data. The filled symbols represent the labeled samples, while the unfilled symbols denote the unlabeled samples. Furthermore, circle symbols denote one class data, while diamond symbols represent the other class data. The dotted lines indicate the best discriminant projection vectors obtained through different algorithms.

FDA and SELF are implemented in the input space. Compared to FDA, SELF utilizes unlabeled data to improve data learning ability. However, as shown in Figure 1a, when dealing with nonlinear data, the performance improvement of SELF is limited. CKFDA and SS-CKFDA are nonlinear extensions of FDA and SELF, respectively. In essence, these implement FDA and SELF in a fused feature space. By nonlinear mapping Φ(X), the sample data in the input space, which is linearly inseparable, becomes separable in the feature space, as shown in Figure 1b. By using the global structure information extracted from the unlabeled data, SS-CKFDA can determine a better discriminant vector that can well-separate the unlabeled data, when compared with CKFDA.

### 2.3. SS-CKFDA for Fault Diagnosis

**Figure 2:**
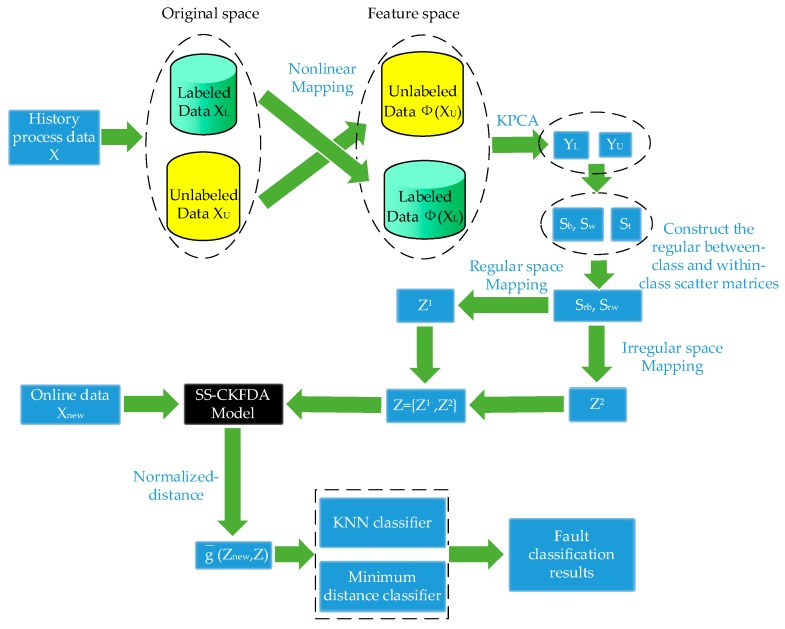
Flowchart of the SS-CKFDA modeling method for online fault diagnosis.

A detail flowchart of the SS-CKFDA modeling method for online fault diagnosis is presented in Figure 2. For the new data sample $x_{new}$, the first step is to extract its discriminant feature $z_{new}$ using the SS-CKPCA model. The next step is to conduct a discriminant analysis based on the discriminant feature $z_{new}$ to determine which fault type the new sample $x_{new}$ belongs to. In the present study, two distance classifiers, KNN classifier and MD classifier, were designed for fault classification.

The KNN classifier, which is a common non-parameter classification method, has extensive research and application background in pattern recognition, machine learning and data mining due to features of being intuitionistic, simple, effective and easy to realize [20]. Its working principle is to first determine the k nearest neighbor of the data sample to be classified in the training set. Then, the data sample is classified by the majority vote of its neighbors. Distance metric is a key part of the KNN classifier, and plays an important role in the performance of the algorithm. In SS-CKFDA, normalized-distance $\bar{g}(z,z_{i})$ defined by Equation (16) is the distance metric. The MD classifier is another simple and easy-to-used distance classifier. First, the mean vector $u_{i}=\left[u_{i}^{1},u_{i}^{2}\right]$ of class i in the training sample is calculated. Then, for the new online data z, if $\bar{g}(z,u_{k})=\min_{i}\bar{g}(z,u_{i})$, then z belongs to class k.

## 3. Experiment Description

In order to verify the performance improvement of fault diagnosis based on the SS-CKFDA algorithm, two experiments that came from different applications are exploited and studied in the present study. The Tennessee Eastman Process (TEP) was employed to evaluate the superiority of the proposed method with other FDA-based algorithms in the viewpoint of less labeled samples situation, and the present method was further validated in the fault diagnosis of an actual automotive assembly process. 

### 3.1. Experiment I: Tennessee Eastman Process

**Figure 3:**
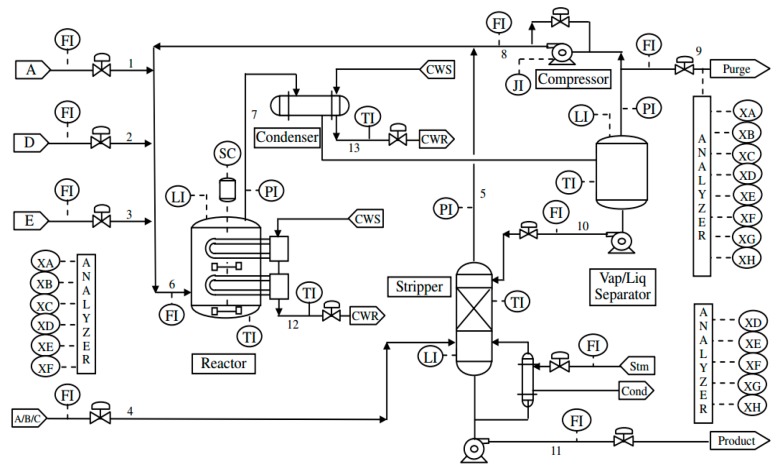
Schematic for the Tennessee Eastman process.

TEP, which has been widely used as a benchmark for evaluating fault diagnosis methods, consists of five main units: a reactor, a condenser, a tripper, a separator, and a compressor [21]. Figure 3 presents the schematic of the TEP. A, C, D and E presents the four gas phase reactants, G and H presents the two liquid products, F presents the byproduct and B presents an inert. The reactor is used to transform the fed gaseous reactants into the liquid products. In the present study, the TEP simulator downloaded from http://www.brahms.scs.uiuc.edu was adopted, which can simulate a normal operating condition and 21 faulty conditions.

In the present study, TEP experimental data consisted of a normal operation mode dataset and four classes of faulty datasets, which were labeled 1–5, respectively. The specific description of a dataset in the present experiment is indicated in Table 1. There were 500 normal operation samples and 100 samples in each type of faulty dataset in the training set. The proportion of labeled data was changed, while the rest of the training samples were unlabeled data. In the present study, the algorithm with a proportion of unlabeled data rates that changed from 10% to 90% was tested. Meanwhile, there are 100 normal operation samples and 200 faulty samples in the testing dataset, with the assumption that the number of samples was equal with the different faulty types. For each sample, all 41 measurement variables were selected as monitored variables.

### 3.2. Experiment II: Automotive Assembly Process

**Figure 4:**
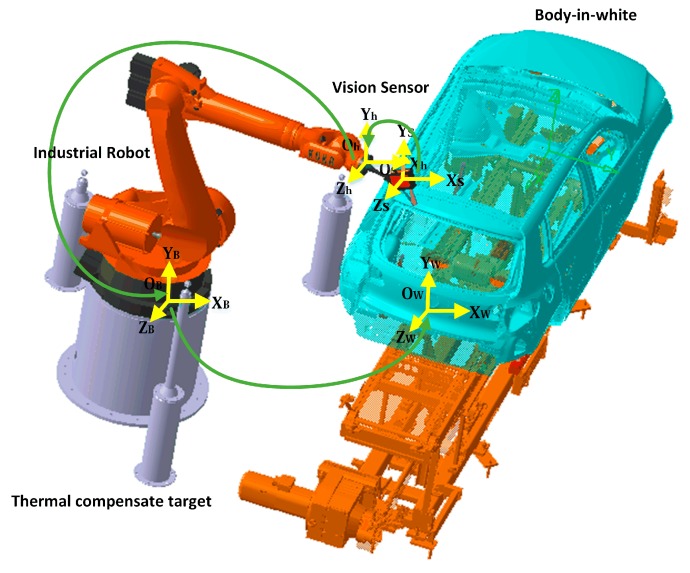
Schematic of the visual inspection system.

As mentioned at the beginning, visual inspection systems have been widely used to monitor the quality of automotive assemblies. As shown in Figure 4, a typical visual inspection system mainly consists of a six-degree-of-freedom industrial robot and a flexible vision sensor [3]. The measuring principle of the vision sensor is the optical triangulation principle [22]. The coordinate systems of the visual inspection system comprise of a workpiece frame ($O_{w}-X_{w}Y_{w}Z_{w}$), a robot base frame ($O_{b}-X_{b}Y_{b}Z_{b}$), an end-effector frame ($O_{h}-X_{h}Y_{h}Z_{h}$) and a vision sensor frame ($O_{s}-X_{s}Y_{s}Z_{s}$). After global calibration was performed for the system, the original coordinate data collected from the vision sensor was unified and accessed in the workpiece frame $O_{w}-X_{w}Y_{w}Z_{w}$.

In the present experiment, the measured values of the rear combination assembly area were chosen to evaluate the proposed fault diagnosis method. The rear combination was the area that was important and more likely to expose the problem. This was assembled by the side panel, floor and trunk lid. In order to eliminate measurement noise, three statistics for each coordinate variables were calculated: the mean value $\bar{x_{i}}=\frac{1}{k}\sum_{j=i-k+1}^{i}\left(x_{j}\right)$, the standard deviation $\sigma_{i}=\sqrt{\frac{1}{k}\sum_{j=i-k+1}^{i}\left(x_{j}-\bar{x_{i}}\right)}$ and the range value $R_{i}=\max\left(x_{i-k+1},x_{i-k+2},\ldots ,x_{i}\right)-\min\left(x_{i-k+1},x_{i-k+2},\ldots ,x_{i}\right)$ with k=5. There were 13 measure coordinates in this area, and 39 features were finally obtained to monitor this assembly. In the rear assembly station, the Nylon blocks on the fixture play an important role in adjusting the position of the auto-body part. Four typical fault modes exist in the nylon blocks deviation: the Y direction deviation on the right side, the X direction deviation on the right side, the Y direction deviation on the left side, and the X direction deviation on the left side. For simplicity, the label Y-R, X-R, Y-L and X-L were used to represent the four fault modes. After a period of data collection and class labeling, a total of 100 samples were acquired for each fault mode. In addition, 100 normal condition samples were also added in the experiment. Similarly, the dataset was divided into the training set and test set to evaluate the performance of various algorithms. 

## 4. Results and Discussion

The SS-CKFDA was used to extract the feature information of two datasets. FDA, SELF and CKFDA were employed to act as controlled trials to demonstrate the validity of the proposed method. Meanwhile, BP-ANN, KNN and SVM were also employed to prove whether SS-CKFDA is able to achieve an ideal performance in dealing with the fault diagnosis in an automotive assembly process.

### 4.1. Parameter Selection Strategy

As described above, the tuning parameter β and kernel parameter σ play important role in SS-CKFDA algorithm. So we analyze the effects of the two parameter separately first. Then we proposed a parameter selection strategy based on repeated grid search cross-validation to find the optimal parameters.

#### 4.1.1. The Effect of Kernel Parameter σ and Tuning Parameter β

**Figure 5:**
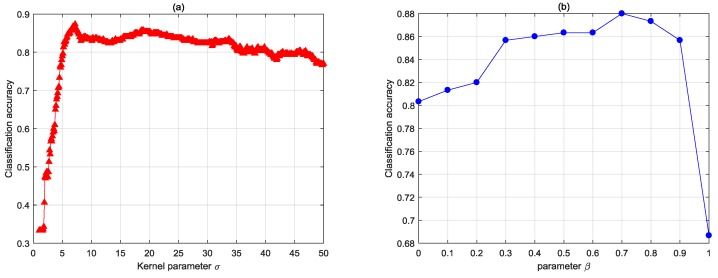
The effect of kernel parameter (**a**) σ and parameter (**b**) β on SS-CKFDA.

First, the effect of the kernel parameter for SS-CKFDA with KNN was evaluated. The other parameters were chosen as follows: the labeled rate and β were set to 0.5; the parameter of KNN is set to 9. Figure 5a presents the performance of SS-CKFDA with the different kernel parameter σ, which ranged from 1 to 50. It was obvious that classification accuracy varies with different kernel values. Generally, the performance content initially increased as a result of the increase in kernel parameters. However, this decreased thereafter when the values of the kernel parameters arrived at a certain stage. 

Then, we evaluated the effect of tuning parameter β with kernel parameter σ set to 7 and the other parameter remained unchanged. Figure 5b shows the performance of SS-CKFDA with the different parameter β ranging from 0 to 1. It is clear that the semi-supervised algorithm outperformed both the unsupervised algorithm when β=0 and the supervised algorithm when β=1. 

It can be observed that the performance of SS-CKFDA algorithms is affected by both the value of the kernel parameter σ and parameter β.

#### 4.1.2. Parameter Selection Strategy

**Figure 6:**
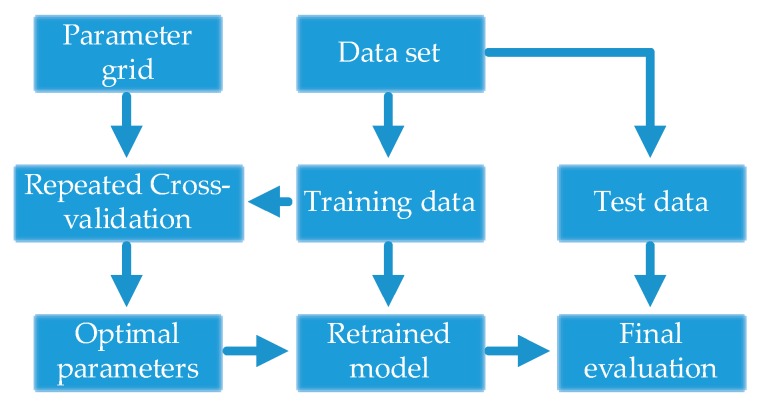
An overview of parameter selection strategy based on repeated grid search cross-validation.

When SS-CKFDA is applied to the TEP data and automotive assembly process data, these two optimization parameters (σ and β) need to be decided. For KNN classifier, the chosen of K is also an important parameter. In this study, we applied cross-validation for parameter tuning. An initial set of possible input parameters were chosen first and then repeated grid search cross-validation algorithm [23] was performed to find optimal parameters for SS-CKFDA. By repeating cross-validation N times and for each grid point generating N cross-validation errors, the tuning parameter with minimal mean cross-validation error was chosen as optimal. An overview of the parameter selection strategy based on repeated grid search cross-validation is presented in Figure 6.

### 4.2. Results of the TEP Data

#### 4.2.1. Selection of Classifier

**Figure 7:**
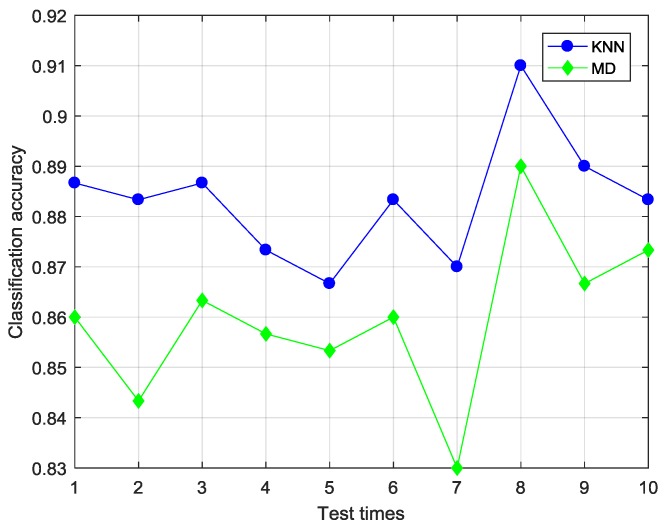
The 10-times test outcome of KNN and MD with the TEP data.

First, the distance classifier to be applied should be determined. SS-CKFDA with KNN and MD was designed to determine which classifier is better for the present situation. The labeled rate was set to 0.5 and other parameters were optimized as follows: β were set to 0.5; the Gaussian kernel parameter σ was set to 7.2 and the parameter of KNN is set to 5. The test ran with 10-fold cross validation for ten times. The average classification accuracy of each time was recorded and shown in Figure 7. The min, max, average and standard deviation statistics were given in Table 2.

It is clear that SS-CKFDA with KNN has better performance than SS-CKFDA with MD. This is because KNN with an optimal k value can be more effectively in avoiding the influence of outliers than MD. So KNN is selected as the major distance classifier for SS-CKFDA.

#### 4.2.2. Results Comparison of SS-CKFDA with Other FDA Algorithms

As a novel FDA based feature extraction algorithm, it is necessary to compare with other FDA algorithms. Thus, we selected FDA, SELF and CKFDA for comparison with different unlabeled rate. The unlabeled rate from 10% to 90% was changed, and the total number of labeled and unlabeled samples was kept constant. Here, we tested 10-fold cross-validation ten times for each unlabeled rate and each algorithm. The average accuracy and standard deviation of each algorithms under different unlabeled rate were listed in Table 3.

**Figure 8:**
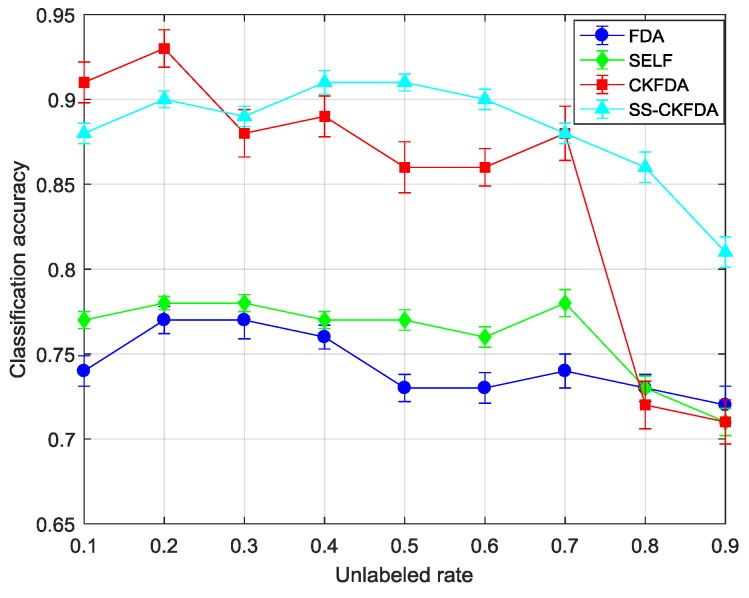
The performance of FDA, SELF, CKFDA and SS-CKFDA with different unlabeled rates on training set.

**Figure 9:**
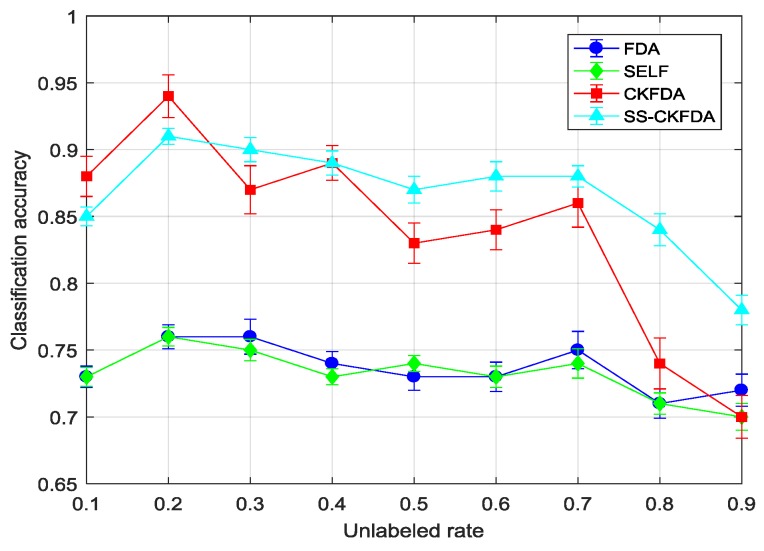
The performance of FDA, SELF, CKFDA and SS-CKFDA with different unlabeled rates on test set.

Table 3 and Figure 8 and Figure 9 present the classification accuracy at different unlabeled rates. FDA and SELF were combined to compare the linear supervised and semi-supervised learning methods. It could be easily observed that FDA and SELF cannot handle this nonlinear data, in which the classification accuracy was the lowest. It was also concluded that SELF cannot use unlabeled data to improve its performance when a nonlinear relationship exists between variables. On the other side, SS-CKFDA can achieve higher accuracy, when compared with CKFDA, as a whole. When the unlabeled rate was relatively low, SS-CKFDA had no advantage, when compared with CKFDA, and CKFDA can even achieve a better performance when the unlabeled rate was 10% and 20%. However, as the unlabeled rate increased, the performance of CKFDA declined more rapidly than SS-CKFDA. When the unlabeled rate reached 80% and 90%, the classification of SS-CKFDA continued to reach to 0.84 and 0.78, respectively, which are significantly superior to CKFDA. This was because as the labeled samples decreased, the model precision decreased for CKFDA. However, for SS-CKFDA, it could use more and more unlabeled samples to correct its model, ensuring its classification accuracy at a high level. On the other hand, it can be seen from the comparison of standard deviation that SS-CKFDA can achieve more stable performance than CKFDA.

### 4.3. Results of the Automotive Assembly Process Data

Similarly, the collected data were divided into the training set and test set. At the same time, a small adjustment was performed, in which the test set was also added to the training procedure as unlabeled data for semi-supervised algorithms. This was because even though the test data was the present prediction data, its measured values remain beneficial for differentiating its fault category. In the present study, 10% of the samples were taken as the training set, and the rest of the samples were used as the test set, which is really consistent with realistic situations. The test ran ten times to reduce accidental error. Furthermore, we selected BP-ANN [24], KNN [20] and SVM [25] for comparison.

For BP-ANN, the parameters were set in this study as follows: three layers (i.e., an input layer, a hidden layer and an output layer) were arranged; the number of neurons in the hidden layer was set 10, the learning rate factor and momentum factor were all set to 0.1; the initial weights were set to 0.3; the learning algorithm was gradient descent backpropagation. For kernel parameter of SVM and k value of KNN, we used the same repeated gird search cross-validation algorithm described in Section 4.1 for parameter selection.

**Figure 10:**
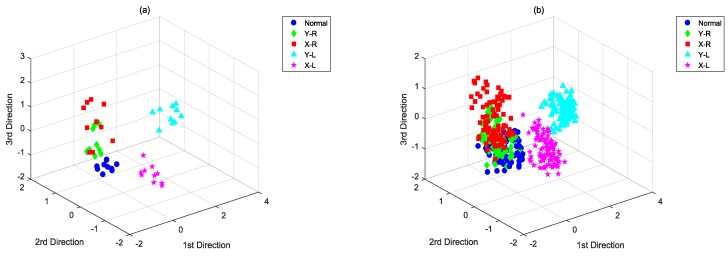
Score plots of SS-CKFDA with the (**a**) training set and (**b**) test set.

Figure 10 demonstrates the score plots of the proposed SS-CKFDA, revealing that the five different kinds of samples were better dispersed. Table 4 lists the results for SS-CKFDA and other classical methods. It was clearly shown that SS-CKFDA can achieve the highest average classification accuracy and lowest standard deviation. For each type of fault, SS-CKFDA always performed better than others. Although SVM has the best performance in the classification of Normal and X_L, it performs poorly in the classification of Y_R and X_R, where SS-CKFDA is significantly better. Summarizing all of the five conditions, SS-CKFDA performs better than SVM and other algorithms.

Furthermore, SS-CKFDA was also compared with other methods with respect to training time, and the results are presented in Table 5. This clearly shows that BP-ANN has the longest training time, while FDA has the least training time. Within these five methods, SS-CKFDA also performs very well. Although the training time was slightly longer than other FDA methods, it can achieve a better accuracy rate when the labeled sample is lesser.

## 5. Conclusions

The present study mainly investigates a novel semi-supervised learning method called, SS-CKFDA, which combines the advantages of semi-supervised learning and CKFDA. The main idea of this method is that the projected vectors extracted from labeled data can be adjusted by integrating the global structure information provided by all labeled and unlabeled samples. We also analyze the effects of the two important parameters β and σ separately and propose a parameter selection strategy based on repeated grid search cross-validation to find the optimal parameters. The experimental results of the TEP data proves that as a novel algorithm, SS-CKFDA coupled with the KNN classifier achieves better and more stable performance, compared to FDA, SELF and CKFDA individually. As the unlabeled rate increases, its advantages become more obvious.

Automotive assembly process data is collected from vision sensors, and the lack of labeled samples is a typical situation. Hence, the proposed method was implied on this. The experimental results of the automotive assembly process data revealed that the new algorithm can be successfully used in automotive manufacturing. When compared with other classical methods, it was observed that when the labeled samples were limited, SS-CKFDA can achieve better average classification accuracy for both training set and test set, allowing training time to satisfy practical use.

## Figures and Tables

**Table 1:** Description of the dataset.

ID	Description	Type	Training Set	Test Set
1	No faulty	/	500	100
2	A/C feed ratio, B composition constant	Step change	100	50
3	B composition, A/C ration constant	Step change	100	50
4	A, B, C feed composition	Random variation	100	50
5	Condenser cooling water inlet temperature	Random variation	100	50

**Table 2:** Outcome of SS-CKFDA with KNN and MD with TEP data.

	Min	Max	Average/Std
SS-CKFDA + KNN	0.87	0.91	0.883/0.012
SS-CKFDA + MD	0.83	0.89	0.859/0.016

**Table 3:** Outcomes of average classification accuracy and standard deviation at different unlabeled rates. (Standard deviations are show in parentheses).

Unlabeled Rate	FDA		SELF		CKFDA		SS-CKFDA	
	Training Set	Test Set	Training Set	Test Set	Training Set	Test Set	Training Set	Test Set
10%	0.74(0.009)	0.73(0.008)	0.77(0.005)	0.73(0.007)	0.91(0.012)	0.88(0.015)	0.88(0.006)	0.85(0.007)
20%	0.77(0.008)	0.76(0.009)	0.78(0.004)	0.76(0.007)	0.93(0.011)	0.94(0.016)	0.9(0.005)	0.91(0.006)
30%	0.77(0.011)	0.76(0.013)	0.78(0.005)	0.75(0.008)	0.88(0.014)	0.87(0.018)	0.89(0.006)	0.90(0.009)
40%	0.76(0.007)	0.74(0.009)	0.77(0.005)	0.73(0.006)	0.89(0.012)	0.89(0.013)	0.91(0.007)	0.89(0.009)
50%	0.73(0.008)	0.73(0.01)	0.77(0.006)	0.74(0.006)	0.86(0.015)	0.83(0.015)	0.91(0.005)	0.87(0.01)
60%	0.73(0.009)	0.73(0.011)	0.76(0.006)	0.73(0.008)	0.86(0.011)	0.84(0.015)	0.9(0.006)	0.88(0.011)
70%	0.74(0.01)	0.75(0.014)	0.78(0.008)	0.74(0.011)	0.88(0.016)	0.86(0.018)	0.88(0.006)	0.88(0.008)
80%	0.73(0.008)	0.71(0.011)	0.73(0.007)	0.71(0.008)	0.72(0.014)	0.74(0.019)	0.86(0.009)	0.84(0.012)
90%	0.72(0.011)	0.72(0.012)	0.71(0.008)	0.70(0.01)	0.71(0.013)	0.70(0.016)	0.81(0.009)	0.78(0.011)
Average	0.744(0.009)	0.737(0.011)	0.761(0.006)	0.732(0.008)	0.849(0.013)	0.839(0.016)	0.882(0.007)	0.867(0.009)

**Table 4:** Outcomes of the average classification accuracy and standard deviation of the automotive assembly process data. (Standard deviations are show in parentheses).

	**FDA**		**SELF**		**CKFDA**		**SS-CKFDA**	
	**Training Set**	**Test Set**	**Training Set**	**Test Set**	**Training Set**	**Test Set**	**Training Set**	**Test Set**
Normal	0.34(0.135)	0.26(0.012)	0.42(0.147)	0.34(0.011)	0.78(0.148)	0.68(0.015)	0.88(0.103)	0.72(0.008)
Y-R	0.56(0.126)	0.55(0.014)	0.72(0.103)	0.52(0.008)	0.66(0.165)	0.53(0.017)	0.82(0.148)	0.66(0.011)
X-R	0.72(0.103)	0.66(0.015)	0.78(0.114)	0.75(0.009)	0.88(0.14)	0.68(0.012)	0.9(0.105)	0.82(0.009)
Y-L	1(0)	1(0)	1(0)	0.98(0.006)	1(0)	1(0)	1(0)	1(0)
X-L	1(0)	0.95(0.011)	1(0)	0.95(0.005)	1(0)	0.95(0.006)	1(0)	0.98(0.003)
Average	0.724(0.073)	0.684(0.01)	0.784(0.072)	0.708(0.008)	0.864(0.091)	0.768(0.01)	0.92(0.071)	0.836(0.006)
	**BP-ANN**		**KNN**		**SVM**			
	**Training Set**	**Test Set**	**Training Set**	**Test Set**	**Training Set**	**Test Set**		
Normal	0.32(0.103)	0.35(0.022)	0.48(0.169)	0.53(0.028)	0.9 (0.141)	0.76(0.011)		
Y-R	0.66(0.135)	0.65(0.025)	0.62(0.148)	0.56(0.025)	0.72(0.193)	0.62(0.014)		
X-R	0.9(0.105)	0.84(0.018)	0.68(0.235)	0.72(0.031)	0.86(0.165)	0.74(0.008)		
Y-L	1(0)	0.95(0.012)	1(0)	1(0)	1(0)	1(0)		
X-L	1(0)	0.95(0.013)	1(0)	0.95(0.019)	1(0)	1(0)		
Average	0.776(0.069)	0.748(0.018)	0.756(0.11)	0.752(0.021)	0.896(0.1)	0.824(0.007)		

**Table 5:** The comparison of average training time.

	Average Training Time (ms)
SS-CKFDA	13.62
CKFDA	12.55
SELF	3.41
FDA	1.60
BP-ANN	265.54
KNN	142.36
SVM	21.78
